# Association Between Ischemic Optic Neuropathy and Inflammatory Bowel Disease: A Population-Based Cohort Study in Taiwan

**DOI:** 10.3389/fmed.2021.753367

**Published:** 2021-09-28

**Authors:** Ting-Yi Lin, Yi-Fen Lai, Po-Huang Chen, Chi-Hsiang Chung, Ching-Long Chen, Yi-Hao Chen, Jiann-Torng Chen, Po-Chen Kuo, Wu-Chien Chien, Yun-Hsiu Hsieh

**Affiliations:** ^1^Department of Ophthalmology, Tri-Service General Hospital, National Defense Medical Center, Taipei City, Taiwan; ^2^Department of Internal Medicine, Tri-Service General Hospital, National Defense Medical Center, Taipei City, Taiwan; ^3^School of Public Health, National Defense Medical Center, Taipei City, Taiwan; ^4^Department of Medical Research, Tri-Service General Hospital, National Defense Medical Center, Taipei City, Taiwan; ^5^Taiwanese Injury Prevention and Safety Promotion Association, Taipei City, Taiwan; ^6^Department of Colon and Rectal Surgery, Tri-Service General Hospital, National Defense Medical Center, Taipei City, Taiwan; ^7^Graduate Institute of Life Sciences, National Defense Medical Center, Taipei City, Taiwan

**Keywords:** chron's disease, extraintestinal manifestation, inflammatory bowel diseases, ischemic optic neuropathy, ocular manifestation, ulcerative colitis

## Abstract

**Background:** Ischemic optic neuropathy (ION) is a possible extraintestinal manifestation (EIM) of inflammatory bowel disease (IBD). We investigate the relation between IBD and ION and possible risk factors associated with their incidence.

**Methods:** Medical records were extracted from the National Health Insurance Research Database (NHIRD) from January 1, 2000, to December 31, 2013. The main outcome was ION development. Univariate and multivariate Cox regression analyses were performed.

**Results:** We enrolled 22,540 individuals (4,508 with IBD, 18,032 without). The cumulative risk of developing ION was significantly greater for patients with IBD vs. patients without (Kaplan–Meier survival curve, *p* = 0.009; log-rank test). Seven (5%) and five (0.03%) patients developed ION in the IBD and control groups, respectively. Patients with IBD were significantly more likely to develop ION than those without IBD [adjusted hazard ratio (HR) = 4.135; 95% confidence interval: 1.312–11.246, *p* = 0.01]. Possible risk factors of ION development were age 30–39 years, diabetes mellitus (DM), hypertension, ischemic heart disease (IHD), atherosclerosis, and higher Charlson comorbidity index revised (CCI_R) value.

**Conclusion:** Patients with IBD are at increased risk of subsequent ION development. Moreover, for patients with comorbidities, the risk of ION development is significantly higher in those with IBD than in those without.

## Introduction

Inflammatory bowel disease (IBD) is a chronic relapsing and remitting intestinal disorder that is typically classified into two subtypes: ulcerative colitis (UC) and Crohn's disease (CD) (1). Both UC and CD are thought to occur in adolescents and adults equally in both genders (2). Ulcerative colitis causes superficial mucosal inflammation of the colon with a contiguous manner of extension. On the other hand, CD is characterized by skip lesions throughout the gastrointestinal tract and transmural inflammation (3).

The highest reported prevalence of IBD is in Europe and North America. In newly industrialized countries in Africa, Asia, and South America, the incidence of IBD has increased substantially since 1990, when these areas became more Westernized (4, 5). In Taiwan, a trend of increasing incidence of IBD (both UC and CD) and decreasing UC-to-CD ratio, especially among those aged 20–39 years, from 2001 to 2015 has been reported (6).

Extraintestinal manifestations (EIMs) of IBD are common and estimated to affect approximately 6% to 47% of adult patients with IBD (7). Although the whole body can be involved, EIMs predominantly affect the skin, joints, biliary tract, and eyes (8). The prevalence of ocular manifestations in IBD range from 0.3 to 13%, based on the population studied, and they occur more frequently in patients with CD (3.5–6%) as compared with UC (1.6–4.6%) (7, 9, 10). The most frequent ocular manifestations observed in IBD patients are episcleritis, scleritis, and uveitis (8, 10). Among ophthalmic EIMs of IBD, some are potentially vision threatening with severe consequences if not treated properly (9–11). In addition, some ocular complications related to IBD treatments have also been reported (8). Ocular complications are usually independent of the extent of the underlying bowel disease and occur in the early years of the disease course (12).

Ischemic optic neuropathy (ION) is one of the possible ophthalmic manifestations of IBD (13). Ischemic optic neuropathy is classified as either anterior ischemic optic neuropathy (AION) or posterior ischemic optic neuropathy (PION), depending on which part of the optic nerve is involved. Anterior ischemic optic neuropathy is more frequent and can be further divided into either arteritic anterior ION, which is often associated with giant cell arteritis, or non-arteritic anterior ischemic optic neuropathy (NAION), which is the most common.

Non-arteritic anterior ischemic optic neuropathy remains the most common cause of acute optic neuropathy among elderly individuals (14). It typically affects patients older than 50 years (mean age at onset ranging from 57 to 65 years) (15), and the incidence has been reported to increase with advancing age (16). Non-arteritic anterior ischemic optic neuropathy causes sudden painless visual loss with positive relative afferent pupillary defect, optic disc edema, and altitudinal visual field defect. Some of the risk factors, such as crowding optic nerve head appearance (small optic nerve head with a small cup-to-disk ratio), diabetes mellitus (DM), systemic hypertension (HTN), hyperlipidemia, and obstructive sleep apnea (OSA) have been reported in the literature (17, 18).

The relation between IBD and ION (especially NAION) has not been well-established in the previous studies. Only a few case reports of the association between ION and CD have been reported. In addition, one of the cases had a personal history of common systemic risk factors related to NAION (13, 19, 20).

Although ION is rare among IBD patients, it has no proven therapy and could cause significant visual impairment in patients. In this study, we aimed to evaluate the relation between IBD and ION as well as the possible risk factors associated with the incidence of ION in patients with IBD.

## Materials and Methods

### Data Source

More than 99% of all of Taiwan's population (including foreigners) are enrolled in the single-payer National Health Insurance program. The National Health Insurance Research Database (NHIRD) of this program possesses registration files and original claim data for reimbursement. The use of NHIRD is limited to research purposes only, and applicants must follow the related laws and regulations of Taiwan.

This retrospective population-based cohort study contained fully anonymized medical records extracted from the NHIRD from January 1, 2000, to December 31, 2013. All patient demographics (including age, gender, index year, related comorbidities) were recorded and analyzed.

### Ethical Considerations

This study was conducted according to the tenets of the Declaration of Helsinki and was approved by the Institutional Review Board of Tri-Service General Hospital (TSGHIRB No. 2-105-05-082). The need for participant informed consent was waived because of the fully anonymized data of NHIRD.

### Patient Selection

We used the International Classification of Disease, Ninth Revision, Clinical Modification (ICD-9-CM) to identify the study population with a diagnosis of IBD (ICD-9-CM code 555, 556). Patients 20 years of age or older who had received medical codes of ICD-9-CM code 555, 556 once during hospitalization or at least three times during outpatient visits were included. Patients with a diagnosis of giant cell arteritis (ICD-9-CM code 446.5) from before 1 year to after 1 year of the index date were excluded. Furthermore, patients who diagnosed as having IBD before January 1, 2000, or with incomplete medical records, defined as incomplete insurance status or unrelated or incorrect given codes, were also excluded.

We selected a group of patients without IBD in the study period as a comparison cohort. This control group was four-fold the number of the IBD group and was matched to the patient's gender, age, and index date. The same exclusion criteria were applied to the control group. Patients were followed until the incidence of ION (ICD-9-CM code 377.41) was found in the records of outpatient or inpatient visits or until the end of the study period (December 31, 2013).

### Comorbidities

We identified the following baseline comorbidities: DM, HTN, hypotension, hyperlipidemia, ischemic heart disease (IHD), atherosclerosis, OSA, renal failure, renal dialysis status, occlusion, and stenosis of the precerebral arteries, occlusion of the cerebral arteries, and hypercoagulable state. In addition, the Charlson comorbidity index revised (CCI_R) was used widely to assess the presence of chronic diseases. Supplementary Table 1 lists the ICD-9-CM codes used in this study for data extraction and analysis.

### Study Outcome

The main outcome was a diagnosis of ION with ICD-9-CM codes (377.41). All subjects were followed from the index date until the occurrence of ION, the date of withdrawal from the insurance system, or the end of 2013.

### Statistical Analysis

We analyzed the characteristics of the patients and compared them between the IBD group and control group (without IBD) in terms of the baseline and the endpoint of this cohort. Categorical variables were reported as numbers and percentages, and continuous variables were reported as means ± standard deviations. Continuous variables in the two groups were compared using independent Student *t*-test; otherwise, Pearson chi-square test and Fisher exact test were used to evaluate the differences in categorical variables. Hazard ratios (HRs) for the association between potential clinical variables, including gender, age group, and related comorbidities, with the development of ION were evaluated using univariate and multivariate Cox regression analyses. We also conducted multivariate analysis of covariance (MANCOVA) to examine possible effects of the cardiovascular-disease-related risk factors as covariant, including DM, occlusion, and stenosis of precerebral arteries, occlusion of cerebral arteries, and hypercoagulable state. Survival outcomes of ION development between the two cohorts were assessed using the Kaplan–Meier method and compared by log-rank test. Statistical significance was defined as *p* < 0.05. All statistical analyses were performed using SPSS software version 22.0 (SPSS Inc., Chicago, IL).

## Results

**Figure 1:**
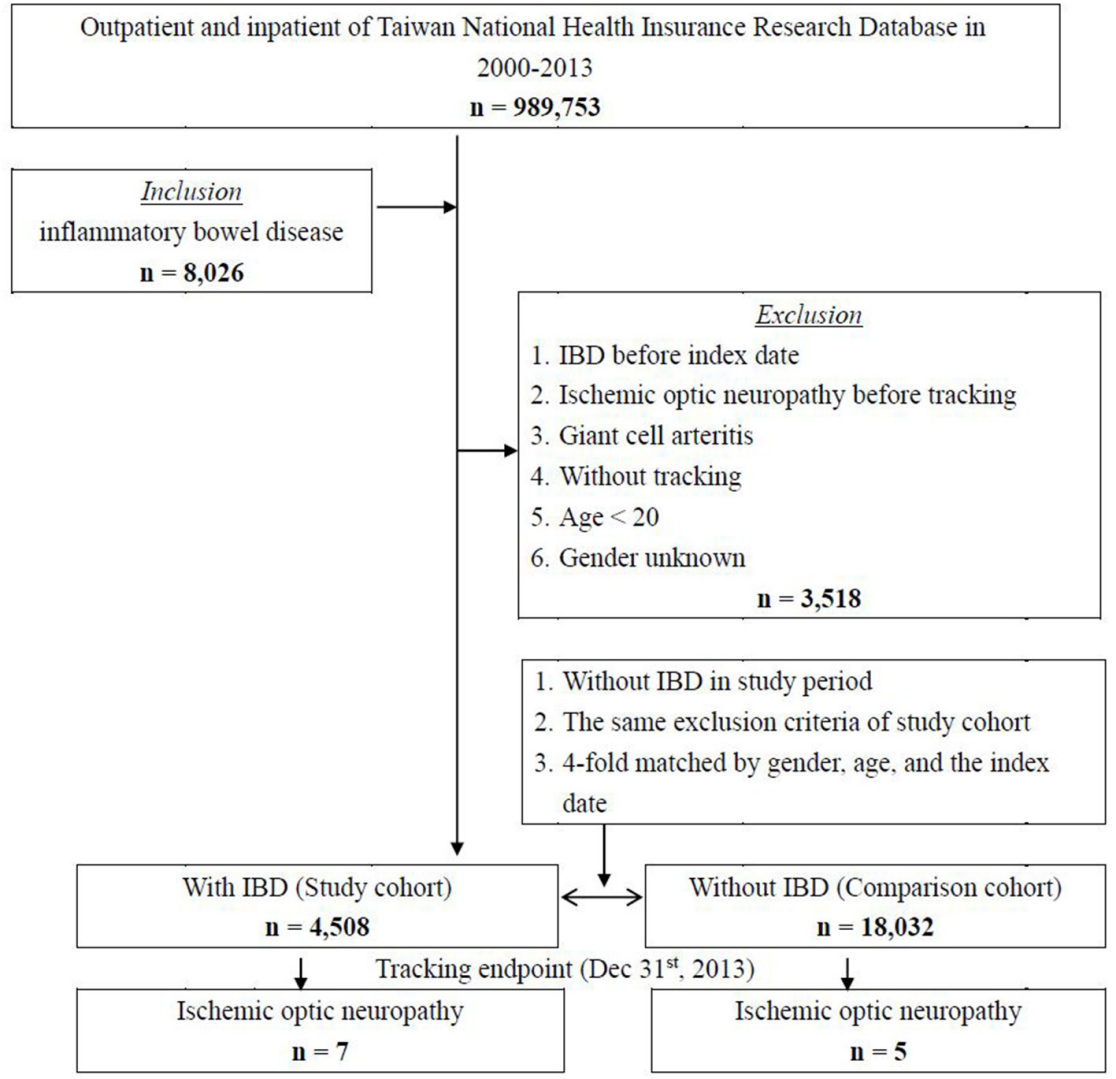
Flowchart of the patient selection in this cohort.

Figure 1 shows the flowchart of the patient selection process used in this cohort. A total of 8,025 patients with IBD were identified from the NHIRD database of 989,753 subjects from 2000 to 2013 in Taiwan. After 3,518 patients were excluded for reasons as listed, a study cohort of 4,508 patients with IBD was selected for further analyses. A four-fold group of 18,032 individuals matched by gender, age, and index date were selected as a control cohort.

### Patients Characteristics

Table 1 lists the demographic characteristics of the enrolled patients at baseline. A total of 22,540 individuals were enrolled in this study, including 4,508 patients with IBD and 18,032 patients without IBD. The mean follow-up period in all patients was 7.53 ± 5.50 years (Supplementary Table 2-1). The mean ages of patients with and without IBD were 55.36 ± 17.45 and 55.07 ± 17.46 years, respectively. Among all age groups, most patients (46.36%) were aged 60 years or older. Considering the related comorbidities, DM, HTN, hypotension, hyperlipidemia, IHD, atherosclerosis, and OSA were found to be significantly increased (all *p* < 0.001) in patients with IBD as compared with patients without IBD. The CCI_R value was also significantly higher in the IBD group (*p* < 0.001).

**Table 1:** The demographic characteristics of the enrolled patients at baseline.

**IBD**	**Total**		**With IBD**		**Without IBD**		
**Variables**	***n***	**%**	***n***	**%**	***n***	**%**	***p***
**Total**	22,540		4,508	20.00	18,032	80.00	
**Gender**							0.999
Male	12,150	53.90	2,430	53.90	9,720	53.90	
Female	10,390	46.10	2,078	46.10	8,312	46.10	
**Age (years)**	55.13 ± 17.45		55.36 ± 17.45		55.07 ± 17.46		0.322
**Age groups (years)**							0.999
20–29	2,240	9.94	448	9.94	1,792	9.94	
30–39	3,275	14.53	655	14.53	2,620	14.53	
40–49	3,375	14.97	675	14.97	2,700	14.97	
50–59	3,200	14.20	640	14.20	2,560	14.20	
≥60	10,450	46.36	2,090	46.36	8,360	46.36	
**DM**							<0.001^**^
Without	18,275	81.08	3,213	71.27	15,062	83.53	
With	4,265	18.92	1,295	28.73	2,970	16.47	
**HTN**							<0.001^**^
Without	18,491	82.04	3,324	73.74	15,167	84.11	
With	4,049	17.96	1,184	26.26	2,865	15.89	
**Hypotension**							<0.001^**^
Without	22,141	98.23	4,310	95.61	17,831	98.89	
With	399	1.77	198	4.39	201	1.11	
**Hyperlipidemia**							<0.001^**^
Without	22,094	98.02	4,273	94.79	17,821	98.83	
With	446	1.98	235	5.21	211	1.17	
**IHD**							<0.001^**^
Without	21,937	97.32	4,327	95.98	17,610	97.66	
With	603	2.68	181	4.02	422	2.34	
**Atherosclerosis**							<0.001^**^
Without	22,378	99.28	4,443	98.56	17,935	99.46	
With	162	0.72	65	1.44	97	0.54	
**OSA**							<0.001^**^
Without	21,369	94.80	4,198	93.12	17,171	95.23	
With	1,171	5.20	310	6.88	861	4.77	
**RF**							0.264
Without	20,511	91.00	4,083	90.57	16,428	91.10	
With	2,029	9.00	425	9.43	1,604	8.90	
**Renal dialysis status**							0.205
Without	20,626	91.51	4,104	91.04	16,522	91.63	
With	1,914	8.49	404	8.96	1,510	8.37	
**Occlusion and stenosis of precerebral arteries**							0.951
Without	22,110	98.09	4,423	98.11	17,687	98.09	
With	430	1.91	85	1.89	345	1.91	
**Occlusion of cerebral arteries**							0.960
Without	22,143	98.24	4,429	98.25	17,714	98.24	
With	397	1.76	79	1.75	318	1.76	
**Hypercoagulable state**							0.680
Without	22,308	98.97	4,459	98.91	17,849	98.99	
With	232	1.03	49	1.09	183	1.01	
**CCI_R**	1.14 ± 1.21		1.4 ± 1.83		1.13 ± 1.19		<0.001^**^

*CCI_R, Charlson comorbidity index revised; DM, diabetes mellitus; HTN, hypertension; IBD, inflammatory bowel disease; IHD, ischemic heart disease; OSA, obstructive sleep apnea; RF, renal failure*.

** *p < 0.001*.

### Outcomes

**Figure 2:**
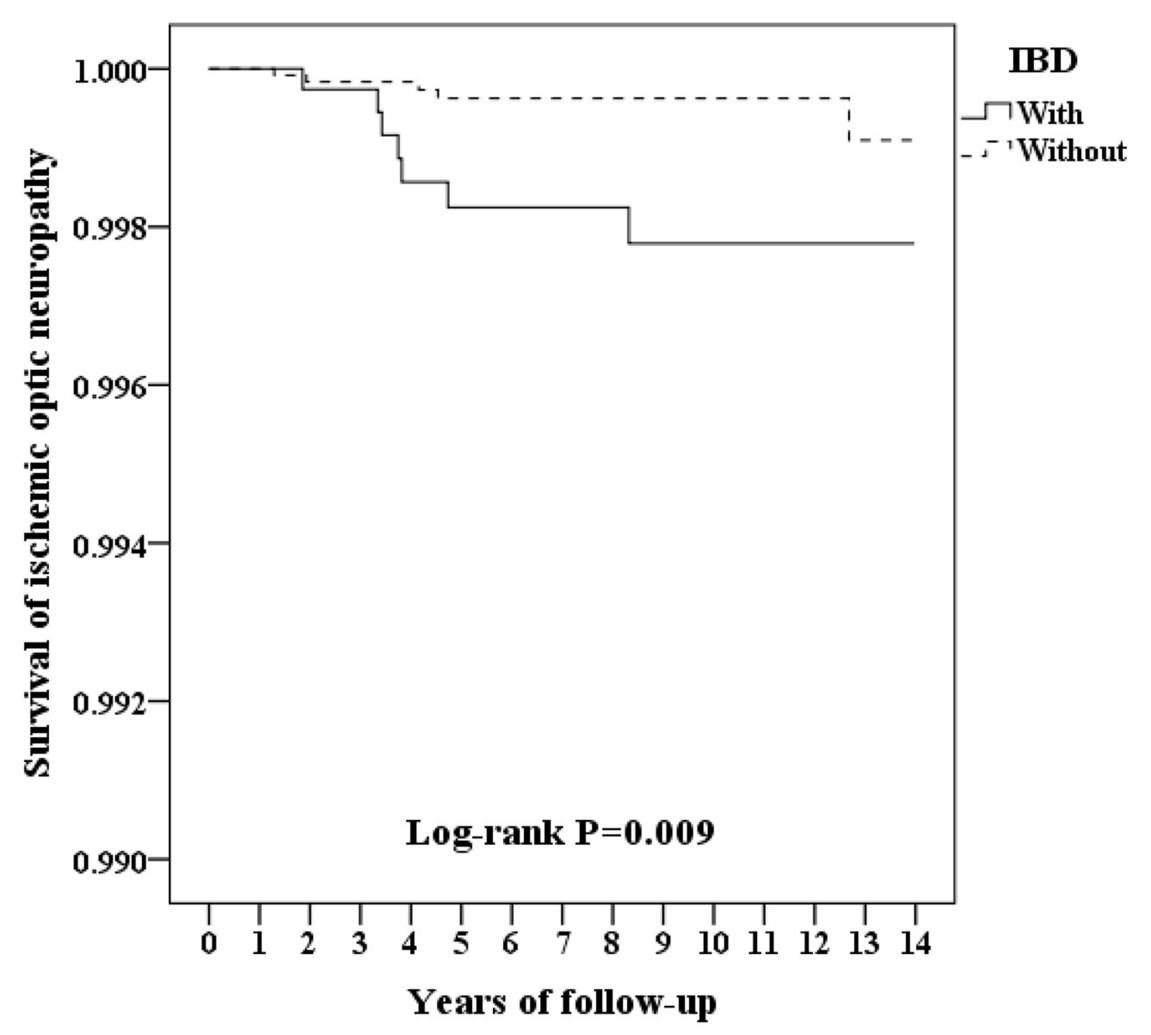
Kaplan–Meier survival curve for ischemic optic neuropathy in patients with and without IBD.

Figure 2 shows the Kaplan–Meier survival curve of the cumulative risk of developing ION in patients with and without IBD. The cumulative risk of ION development was significantly greater for patients with IBD vs. patients without IBD (*p* = 0.009; log-rank test). The mean survival time to ION development was 4.18 ± 2.02 years in patients with IBD and 4.92 ± 4.56 years in patients without IBD (Supplementary Table 2-2).

After we adjusted for confounding factors, multivariate analysis using Cox regression demonstrated that patients with IBD had a significantly higher probability of developing ION than did patients without IBD (adjusted HR = 4.135; 95% CI: 1.312–11.246, *p* = 0.01; Table 2). For other potential clinical variables evaluated by Cox regression analyses, age between 30 and 39 years; patients with underlying comorbidities of DM, HTN, IHD, or atherosclerosis; and patients with higher CCI_R score had a significantly higher probability of developing ION (Table 2). The cardiovascular-disease-related risk factors were entered as covariant into a MANCOVA analysis, which revealed IBD was statistically significantly associated with the incidence of ION (Wilks' lambda = 0.125, *F* = 2.709, *p* = 0.003), confirming in consistent with our result.

**Table 2:** Risk analysis for ischemic optic neuropathy.

**Variables**	**Crude HR (95% CI)**	***p***	**Adjusted HR (95% CI)**	***p***
**IBD**				
Without	Reference		Reference	
With	4.795 (1.865–15.124)	0.009^*^	4.135 (1.312–11.246)	0.01^*^
**Gender**
Male	3.101 (0.802–11.452)	0.165	2.935 (0.728–10.846)	0.112
Female	Reference		Reference	
**Age groups (years)**				
20–29	0	0.997	0	0.998
30–39	5.121 (1.165–19.987)	0.011^*^	4.866 (1.195–19.834)	0.011^*^
40–49	0.972 (0.106–7.121)	0.845	0.826 (0.097–6.923)	0.875
50–59	1.571 (0.365–7.386)	0.623	1.483 (0.347–7.301)	0.632
≥60	Reference		Reference	
**DM**				
Without	Reference		Reference	
With	3.301 (1.898–5.449)	<0.001^**^	3.184 (1.688–5.010)	<0.001^**^
**HTN**				
Without	Reference		Reference	
With	3.597 (1.798–5.454)	<0.001^**^	3.215 (1.550–5.267)	<0.001^**^
**Hypotension**				
Without	Reference		Reference	
With	2.987 (0.895–4.985)	0.895	1.735 (0.712–4.628)	0.489
**Hyperlipidemia**				
Without	Reference		Reference	
With	2.563 (0.597–5.986)	0.298	2.234 (0.411–5.309)	0.387
**IHD**				
Without	Reference		Reference	
With	4.896 (2.267–6.891)	<0.001^**^	3.251 (1.862–5.703)	<0.001^**^
**Atherosclerosis**				
Without	Reference		Reference	
With	2.450 (1.885–2.890)	<0.001^**^	1.568 (1.007–2.037)	0.042^*^
**OSA**				
Without	Reference		Reference	
With	3.896 (1.070–5.992)	0.003^*^	2.790 (0.873–4.286)	0.124
**RF**				
Without	Reference		Reference	
With	1.896 (0.756–2.616)	0.344	1.924 (0.833–2.784)	0.288
**Renal dialysis status**				
Without	Reference		Reference	
With	1.903 (0.770–2.865)	0.398	1.976 (0.845–2.975)	0.291
**Occlusion and stenosis of precerebral arteries**				
Without	Reference		Reference	
With	2.104 (1.066–3.187)	0.018^*^	1.493 (0.867–2.088)	0.206
**Occlusion of cerebral arteries**				
Without	Reference		Reference	
With	2.075 (1.037–3.089)	0.029^*^	1.465 (0.812–1.999)	0.181
**Hypercoagulable state**				
Without	Reference		Reference	
With	6.832 (0.096–17.154)	0.957	3.045 (0.039–12.187)	0.976
**CCI_R**	1.454 (1.112–1.678)	<0.001^*^	1.326 (1.104–1.638)	<0.001^**^

*Adjusted HR means adjusted for variables listed in the table. CCI_R, Charlson comorbidity index revised; DM, diabetes mellitus; HR, hazard ratio; HTN, hypertension; IBD, inflammatory bowel disease; IHD: ischemic heart disease, OSA, obstructive sleep apnea; RF, renal failure*.

* *p < 0.05*.

** *p < 0.001*.

We further conducted stratified subgroup analyses by multivariate Cox regression; the results are provided in Table 3. At the study endpoint, seven (5%) patients with IBD and five (0.03%) patients without IBD developed ION. Supplementary Table 3 shows the detailed information of IBD and Non-IBD patients who developed ION in this cohort. The overall incidence rate of ION was 17.7 per 100,000 person-years in patients with IBD and 4.28 per 100,000 person-years in patients without IBD. Male patients with IBD had a significantly higher risk of ION development as compared with male patients without IBD (adjusted HR = 6.203; 95% CI: 1.951–19.678, *p* < 0.001). For patients aged 30–39 years and ≥60 years, IBD was significantly associated with risks of developing ION (adjusted HR = 8.812; 95% CI: 2.634–25.039; *p* < 0.001; and adjusted HR = 5.993; 95% CI: 1.743–17.572; *p* < 0.001, respectively). Inflammatory bowel disease also significantly increased the risk of ION development, whether or not patients had DM, occlusion and stenosis of the precerebral arteries, occlusion of the cerebral arteries, and hypercoagulable state. Nevertheless, the significance was more prominent in patients with these comorbidities as compared with patients without these comorbidities. Among all other clinical variables, the adjusted HRs were significantly higher in IBD patients with HTN, hypotension, IHD, atherosclerosis, OSA, renal failure, and renal dialysis status (adjusted HR = 9.25, 4.137, 5.083, 4.288, 4.138, 5.713, and 4.487, respectively; all *p* < 0.001).

**Table 3:** Risk analysis for ischemic optic neuropathy stratified by demographic and clinical characteristics between patients with/without IBD.

	**With IBD**		**Without IBD**		**Ratio**	**With vs. Without** ***(Reference)***	
**Strarified**	**Events**	**Rate (per 10^**5**^ PYs)**	**Events**	**Rate (per 10^**5**^ PYs)**		**Adjusted HR (95% CI)**	***P***
**Total**	7	17.70	5	4.28	4.132	4.135 (1.312–11.246)	0.01^*^
**Gender**							
Male	6	29.30	3	4.82	6.081	6.203 (1.951–19.678)	<0.001^**^
Female	1	5.25	2	3.68	1.428	1.448 (0.442–4.535)	0.794
**Age groups (years)**							
20–29	0	0.00	0	0.00	–	–	–
30–39	3	110.85	1	12.63	8.778	8.812 (2.634–25.039)	<0.001^**^
40–49	1	17.60	0	0.00	∞	∞	0.993
50–59	0	0.00	2	11.65	0	0.000	0.987
≥60	4	10.19	2	1.73	5.885	5.993 (1.743–17.572)	<0.001^**^
**DM**							
Without	4	13.57	4	4.29	3.167	3.231 (1.010–10.084)	0.041^*^
With	3	29.81	1	4.28	6.960	6.973 (2.226–21.172)	<0.001^**^
**HTN**							
Without	3	9.89	4	4.25	2.328	2.276 (0.733–7.381)	0.759
With	4	43.48	1	4.44	9.791	9.250 (2.184–26.613)	<0.001^**^
**Hypotension**							
Without	4	10.53	4	3.48	3.029	3.106 (0.862–7.265)	0.288
With	3	195.00	1	63.30	3.081	4.137 (1.318–11.250)	<0.001^**^
**Hyperlipidemia**							
Without	3	7.95	5	4.35	1.830	1.862 (0.573–5.872)	0.649
With	4	219.06	0	0.00	∞	∞	0.909
**IHD**							
Without	4	10.54	4	3.51	3.002	3.020 (0.952–8.010)	0.068
With	3	188.96	1	36.41	5.189	5.083 (1.597–12.206)	<0.001^**^
**Atherosclerosis**							
Without	3	7.70	4	3.45	2.234	2.235 (0.721–5.872)	0.287
With	4	692.96	1	159.27	4.351	4.288 (1.367–11.450)	<0.001^**^
**OSA**							
Without	3	8.15	3	2.70	3.020	3.029 (0.956–8.024)	0.061
With	4	147.08	2	36.45	4.035	4.138 (1.381–11.973)	<0.001^*^
**RF**							
Without	1	2.79	2	1.89	1.479	1.483 (0.496–4.259)	0.511
With	6	160.96	3	27.91	5.767	5.713 (1.732–13.67)	<0.001^**^
**Renal dialysis status**							
Without	2	5.56	2	1.87	2.971	3.154 (0.986–7.297)	0.059
With	5	140.91	3	30.70	4.591	4.487 (1.426–12.035)	<0.001^**^
**Occlusion and stenosis of precerebral arteries**							
Without	5	12.89	4	3.50	3.685	3.875 (1.086–10.007)	0.011^*^
With	2	264.47	1	42.72	6.191	6.020 (1.923–15.386)	<0.001^**^
**Occlusion of cerebral arteries**							
Without	4	10.30	3	2.62	3.935	3.828 (1.042–9.241)	0.033^*^
With	3	432.77	2	97.19	4.453	4.456 (1.436–12.185)	<0.001^**^
**Hypercoagulable state**							
Without	5	12.78	4	3.46	3.692	3.691 (1.000–8.096)	0.05^*^
With	2	466.13	1	84.38	5.524	5.529 (1.786–14.204)	0.006^*^

*Adjusted HR means adjusted for variables listed in the table. CCI_R, Charlson comorbidity index revised; DM, diabetes mellitus; HR, hazard ratio; HTN, hypertension; IBD, inflammatory bowel disease; IHD, ischemic heart disease; OSA, obstructive sleep apnea; PYs, person-years; RF, renal failure, ∞ indicates infinity*.

* *p < 0.05*.

** *p < 0.001*.

## Discussion

We conducted a 13-year follow-up population-based study using data obtained from the Taiwan NHIRD. To our knowledge, this is the first cohort study to investigate the associations between ION and IBD and to evaluate the possible risk factors in the incidence of ION in IBD patients. As compared with patients without IBD, those with IBD had a statistically significant higher risk of developing ION. In addition, age 30–39 years; presence of DM, HTN, IHD, or atherosclerosis; and higher CCI_R scores were identified as significant risk factors for the development of ION.

The etiopathogenesis of NAION is controversial and still debated (21). The disease entity is considered to be an acute ischemic insult of the optic nerve head secondary to low blood flow in the posterior ciliary arteries (13, 21, 22). Multiple factors may correlate with the incidence of NAION, including local predisposing factors (e.g., crowding optic nerve head appearance, optic disc drusen), precipitating factors (e.g., severe hypotension, impaired autoregulation of the disc circulation, posterior vitreous detachment, general anesthesia, or dialysis), and systemic vascular diseases (e.g., OSA, HTN, DM, hyperlipidemia) (21, 23). In this study, we found a significantly higher risk of developing ION in IBD patients after adjusting for confounding factors. The interplay between ION and IBD remains unclear. It might be multifactorial and requires further investigation. Some theories have been proposed to explain the pathophysiological mechanisms of ophthalmic complications of IBD, including vaso-occlusive phenomena (24), action of antigen–antibody complexes produced against the vessels of the bowel wall (9), occlusion secondary to inflammation or hypercoagulability (22), and side effects related to IBD treatment (8, 25, 26).

Some possible risk factors of ION development were identified by multivariate Cox regression in this cohort, including age 30–39 years; presence of DM, HTN, IHD, or atherosclerosis; and higher CCI_R score. Ambiguities exist in studies regarding the possible association between gender and ophthalmic manifestations of IBD. Bernstein et al. described the association between female sex and ocular manifestation in IBD, but only iritis and uveitis were analyzed in that study (27). According to the results of our study (although not significant) and previous case reports of ION in IBD (in which all patients were male) (13, 19, 20), there may be a male predilection for ION development in IBD patients. Patients aged 30–39 years showed a significantly higher risk of ION development. This age peak in the incidence of ION might be due to the fact that IBD patients tend to develop at least one EIM within 15 years of disease diagnosis, and most are diagnosed in their 20s to 30s (28). Among all other possible risk factors, most are correlated with systemic vascular diseases. Because ION is presumed to be a vascular disease of the optic nerve head (29), the presence of these comorbidities in IBD patients may imply some synergistic effects on ION development. Nevertheless, further study is needed to elucidate their relation and underlying mechanisms.

There are several strengths of this study. First, this was a nationwide population-based cohort study from a million-level database in Taiwan. Because of its large sample size and the long-term follow-up period, the study provided relatively good statistical power for this rare disease in IBD patients. Second, a four-fold control group matched by gender, age, and index date was selected for comparison. In addition, possible confounding factors were adjusted during multivariate Cox regression analysis.

However, several study limitations should be noted. First, nearly all study participants were from the Taiwan population, thus preventing generalization and the application of the study results to patients from other racial or ethnic groups. A study conducted in the United States concluded that Asians (US-born Asians and Asian immigrants) are more likely to have ocular EIM as compared with White patients (3.4 vs. 0.7%, *p* = 0.022) (30). In contrast, another systemic review showed that no major differences in EIM between African Americans, Hispanics, and Asians (31).

Another limitation is that the medications prescribed (either for IBD or other diseases) were not stratified in this study, and consequently, the influence of medication treatment could not be ruled out. There is no ultimate treatment of IBD and the treatment-related side effects should be considered individually. Among all of the current treatments of IBD, there was no reported correlation with ION (32, 33). Either treatment-related complications or possible protective effects of IBD drugs toward the ION development need further studies to investigate.

To date, there are no definitive, proven treatments or prophylaxes for NAION (21, 34, 35). Because most of the NAION patients have one or more vascular risk factors (18, 36), controlling these systemic risk factors remains an important issue and is always suggested. Moreover, the stage and severity of the underlying diseases might have some influence on the hazard of developing NAION (37). Third, the NHIRD database does not provide blood test and imaging data such as magnetic resonance imaging or fundus images. The ION could be identified only by the ICD-9-CM codes as described. As a result, the possibility of misdiagnosis cannot be ruled out in this study. Finally, we did not analyze the influence of other ocular manifestations and treatment-related ocular complications on the incidence of ION in IBD patients.

In conclusion, patients with IBD are at increased risk of subsequent ION development during follow-up in this cohort. In addition, among patients with some comorbidities, especially systemic vascular risk factors, the risks of ION development were significantly higher in those with IBD than in those without IBD. It is important for the clinician to remain vigilant regarding visual complaints in IBD patients. If IBD patients present with systemic vascular risk factors, these factors should be properly treated. Furthermore, regular ophthalmic follow-up in IBD patients, especially those with higher risk, may be of great importance.

## Data Availability Statement

The original contributions presented in the study are included in the article/Supplementary Material, further inquiries can be directed to the corresponding author/s.

## Ethics Statement

The studies involving human participants were reviewed and approved by Institutional Review Board of Tri-Service General Hospital. Written informed consent for participation was not required for this study in accordance with the national legislation and the institutional requirements.

## Author Contributions

T-YL, W-CC, and Y-HH: conceptualization and validation. T-YL, Y-FL, P-HC, C-HC, P-CK, and W-CC: methodology. T-YL and Y-HH: literature search. C-HC and W-CC: data acquisition, statistical analysis, and data curation. T-YL, Y-FL, P-HC, C-HC, W-CC, P-CK, and Y-HH: data analysis and investigation. T-YL: manuscript preparation. P-HC, C-HC, C-LC, Y-HC, J-TC, W-CC, and Y-HH: manuscript editing. C-HC, C-LC, Y-HC, J-TC, P-CK, W-CC, and YHH: supervision. Y-HH and W-CC: project administration and funding acquisition. All authors have read and agreed to the published version of the manuscript.

## Funding

This study was supported by the Tri-Service General Hospital Research Foundation (TSGH-B-110012 and TSGH-D-110110), and the sponsor has no role in study design, data collection and analysis, decision to publish, or preparation of the manuscript.

## Conflict of Interest

The authors declare that the research was conducted in the absence of any commercial or financial relationships that could be construed as a potential conflict of interest.

## Publisher's Note

All claims expressed in this article are solely those of the authors and do not necessarily represent those of their affiliated organizations, or those of the publisher, the editors and the reviewers. Any product that may be evaluated in this article, or claim that may be made by its manufacturer, is not guaranteed or endorsed by the publisher.
