# Supplementary material for: Association Between Ischemic Optic Neuropathy and Inflammatory Bowel Disease: A Population-Based Cohort Study in Taiwan
Source: Front Med (Lausanne). 2021 Sep 28;8:753367. doi: 10.3389/fmed.2021.753367 (PMC8509714; doi:10.3389/fmed.2021.753367)
Supplement: Supplementary file 1 [file Table_1.DOCX]

Supplementary Material

| **Table S1.** ICD-9-CM codes used in this study for data extraction and analysis. | |
| --- | --- |
|  | **ICD-9-CM / definition** |
| **Stduy population:** Inflammtory bowel disease (IBD) | ≧3 outpatient visits or ≧1 inpatient |
| Crohn's disease | 555 |
| Ulcerative colitis | 556 |
| **Excluding:** Giant cell arteritis | 446.5; From before 1 year to after 1 year of index date |
| **Events:** Ischemic optic neuropathy (ION) | 377.41 |
| **Comorbidities:** | In study period |
| Diabetes mellitus (DM) | 250 |
| Hypertension (HTN) | 401-405 |
| Hypotension | 458 |
| Hyperlipidemia | 272 |
| Ischemic heart disease (IHD) | 410-414 |
| Atherosclerosis | 440 |
| Obstructive sleep apnea (OSA) | 327.23 |
| Renal failure (RF) | 586 |
| Renal dialysis status | V45.11 |
| Occlusion and stenosis of precerebral arteries | 433 |
| Occlusion of cerebral arteries | 434 |
| Hypercoagulable state | 289.81-289.82 |
| **Charlson comorbidity index revised (CCI_R)** | CCI removed DM, HTN, IHD, RF, occlusion and stenosis of precerebral arteries, and occlusion of cerebral arteries |

| **Table S2-1.** Years of follow-up | | | | |
| --- | --- | --- | --- | --- |
| **IBD** | **Min** | **Median** | **Max** | **Mean ± SD** |
| With | 0.01 | 6.23 | 13.97 | 7.77 ± 5.16 |
| Without | 0.01 | 5.72 | 14.00 | 7.47 ± 5.58 |
| Overall | 0.01 | 5.91 | 14.00 | 7.53 ± 5.50 |

| **Table S2-2.** Years to ischemic optic neuropathy | | | | |
| --- | --- | --- | --- | --- |
| **IBD** | **Min** | **Median** | **Max** | **Mean ± SD** |
| With | 1.86 | 3.75 | 8.32 | 4.18 ± 2.02 |
| Without | 1.29 | 4.16 | 12.68 | 4.92 ± 4.56 |
| Overall | 1.29 | 3.78 | 12.68 | 4.49 ± 3.15 |

| **Table S3.** Clinical features of patients who developed ischemic optic neuropathy in this cohort | | | | | | | | | | | | |
| --- | --- | --- | --- | --- | --- | --- | --- | --- | --- | --- | --- | --- |
| **Patient** | 1 | 2 | 3 | 4 | 5 | 6 | 7 | 8 | 9 | 10 | 11 | 12 |
| IBD | With | | | | | | | Without | | | | |
| Gender | Male | Male | Male | Male | Female | Male | Male | Male | Male | Female | Male | Female |
| Age (yrs) | 30.26 | 33.78 | 38.54 | 43.86 | 61.25 | 68.83 | 71.01 | 35.56 | 54.86 | 57.73 | 67.84 | 72.20 |
| DM | Without | Without | Without | With | With | With | Without | Without | Without | Without | With | Without |
| HTN | Without | Without | Without | With | With | With | Without | Without | Without | Without | Without | With |
| Hypotension | Without | With | Without | With | Without | With | Without | Without | Without | Without | Without | With |
| Hyperlipidemia | Without | Without | With | Without | With | With | With | Without | Without | Without | Without | Without |
| IHD | With | Without | With | Without | Without | With | Without | Without | Without | With | Without | Without |
| Atherosclerosis | Without | Without | With | With | With | With | Without | With | Without | Without | Without | Without |
| OSA | With | With | With | Without | With | Without | Without | With | Without | With | Without | Without |
| RF | Without | With | With | With | With | With | With | With | With | With | Without | Without |
| Renal dialysis status | Without | Without | With | With | With | With | With | Without | Without | With | With | With |
| Occlusion and stenosis of precerebral arteries | With | Without | Without | With | Without | Without | Without | With | Without | Without | Without | Without |
| Occlusion of cerebral arteries | Without | With | Without | With | With | Without | Without | Without | With | Without | With | Without |
| Hypercoagulable state | Without | Without | With | With | Without | Without | Without | Without | Without | Without | Without | With |
| CCI_R | 0 | 3 | 1 | 1 | 0 | 4 | 6 | 0 | 1 | 4 | 1 | 6 |
| Note: CCI_R: Charlson comorbidity index revised, DM: Diabetes Mellitus, HTN: hypertension, IBD: Inflammatory Bowel Disease, IHD: Ischemic Heart Disease, OSA: Obstructive Sleep Apnea, RF: Renal Failure. | | | | | | | | | | | | |
